# Supplementary figures and images for: Necrotic debris and STING exert therapeutically relevant effects on tumor cholesterol homeostasis
Source: Life Sci Alliance. 2022 Jan 4;5(3):e202101256. doi: 10.26508/lsa.202101256 (PMC8742871; doi:10.26508/lsa.202101256)

**Figure 4C**

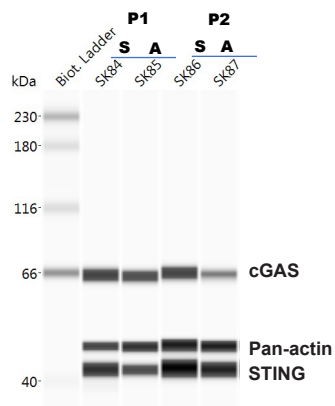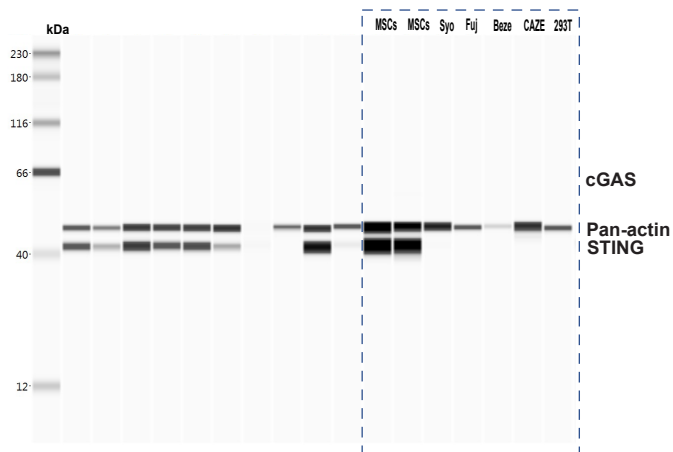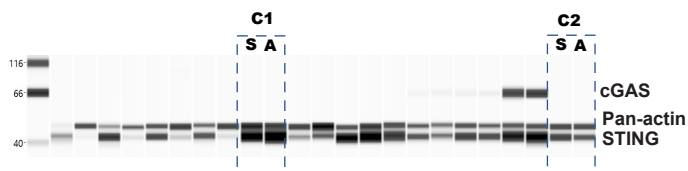

Supplement: Supplementary file 1 [file LSA-2021-01256_SdataF4.1.pdf]

Figure 4D

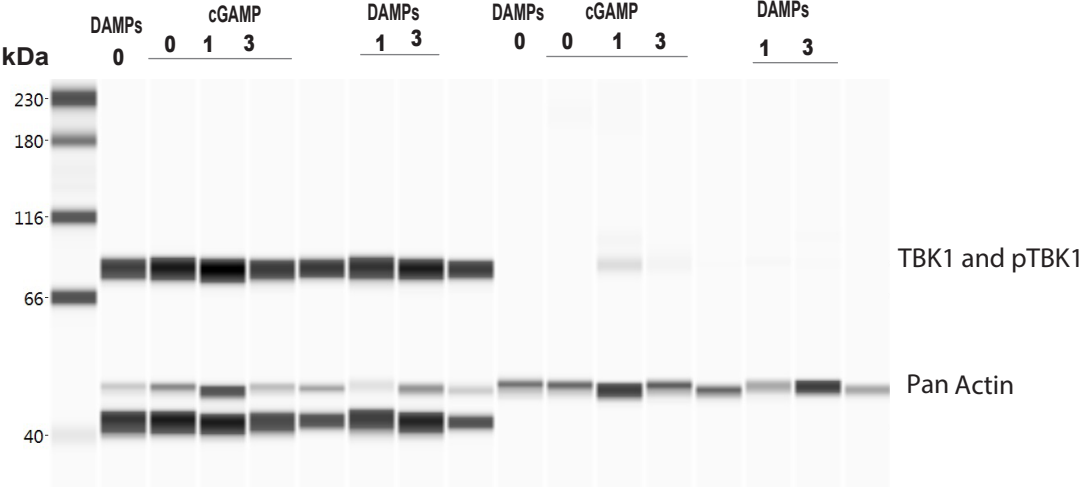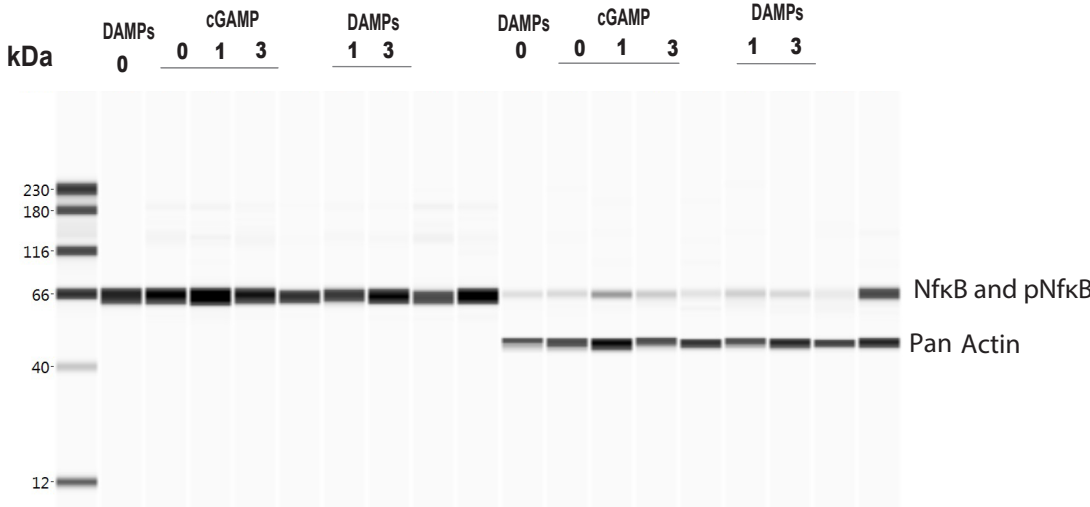

Supplement: Supplementary file 2 [file LSA-2021-01256_SdataF4.2.pdf]

Figure 4D

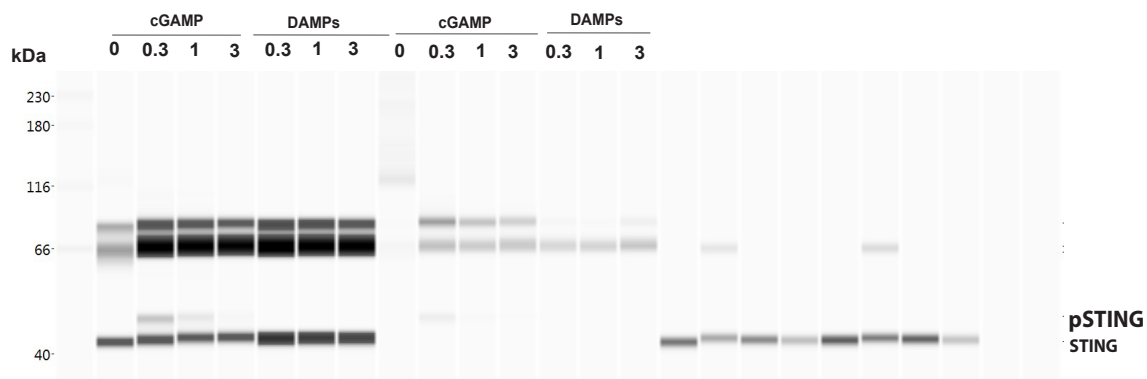

Supplement: Supplementary file 3 [file LSA-2021-01256_SdataF4.3.pdf]

Figure S7A

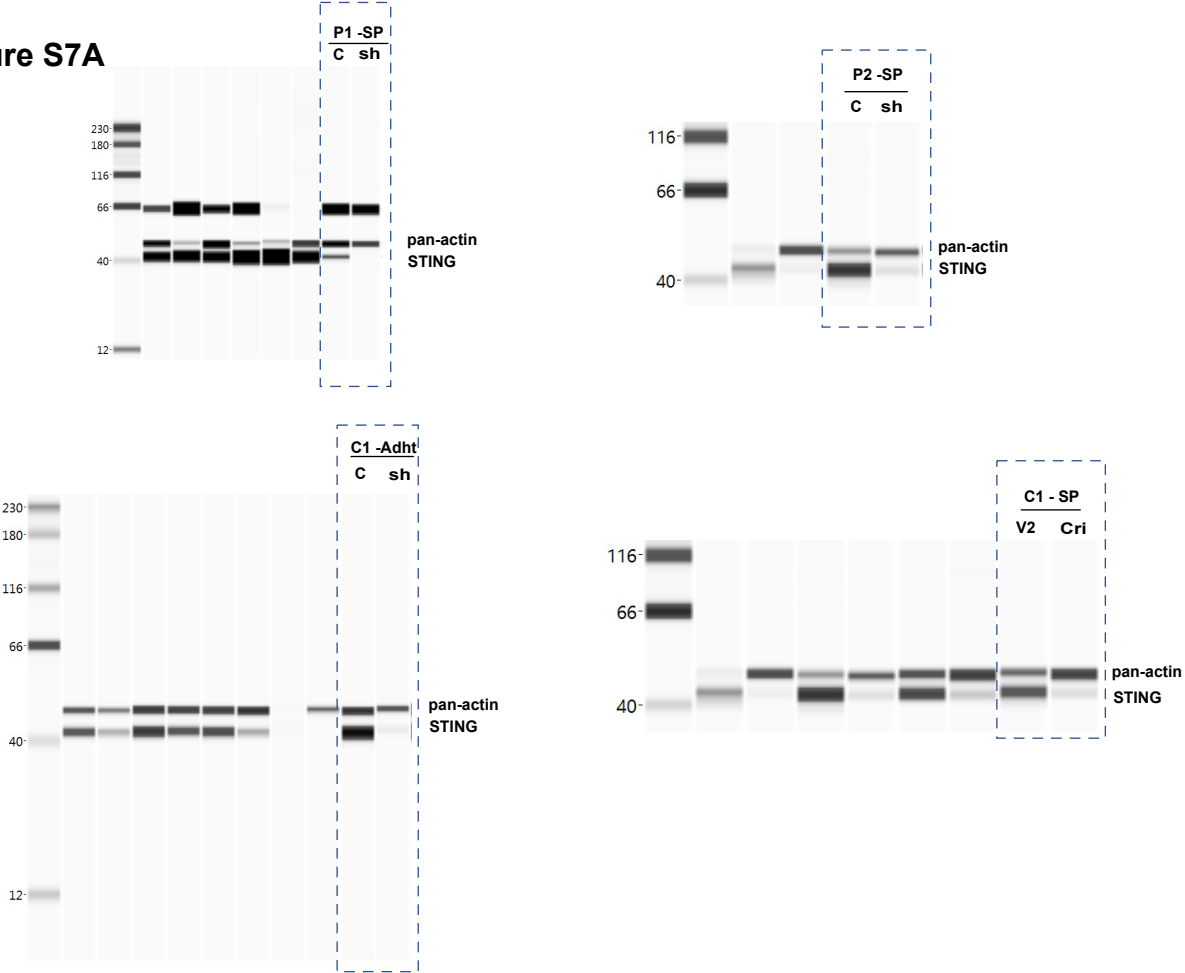

Supplement: Supplementary file 4 [file LSA-2021-01256_SdataFS7.pdf]
