# Supplementary material for: Necrotic debris and STING exert therapeutically relevant effects on tumor cholesterol homeostasis
Source: Life Sci Alliance. 2022 Jan 4;5(3):e202101256. doi: 10.26508/lsa.202101256 (PMC8742871; doi:10.26508/lsa.202101256)
Supplement: Supplementary file 5 [file LSA-2021-01256_TableS1.docx]

| **Genes** | **Primers** |  |
| --- | --- | --- |
| ***GAPDH*** | FW 5’ GGC TCT CCA GAA CATCAT CC 3’ |  |
|  | RV 5’ CCT GCT TCA CCA CCT TCT TG 3’ |  |
| ***TLR1*** | FW 5’ CAAATGGAACAGACAAGCAGG 3’ |  |
|  | RV 5’ GCCTGGTACCCCTATTAGTG 3’ |  |
| ***TLR2*** | FW 5’ AGACCTATAGTGACTCCCAGG 3’ |  |
|  | RV 5’ ACCCACACCATCCACAAAG 3’ |  |
| ***TLR3*** | FW 5’ TCAACTTTCTGATAAAACCTTTGCC 3’ |  |
|  | RV 5’ AGATGACAAGCCATTATGAGACA 3’ |  |
| ***TLR4*** | FW 5’ TGCGTGAGACCAGAAAGC 3’ |  |
|  | RV 5’ TTAAAGCTCAGGTCCAGGTTC 3’ |  |
| ***TLR5*** | FW 5’ TCCAGGGTTCAAGCGATTC 3’ |  |
|  | RV 5’ CGTTGTCAGTAGCATCAGGAG 3’ |  |
|  | FW 5’ TGGACTCATATCAAGATGCTCTG 3’ |  |
|  | RV 5’ GTCGGAGAACTGGATTCTGG 3’ |  |
| ***TLR7*** | FW 5’ GAAAGTTGATGCTATTGGGCC 3’ |  |
|  | RV 5’ GAATTTGTCTCTTCAGTGTCCAC 3’ |  |
| ***TLR8*** | FW 5’ CTGCATAGAGGGTACCATTCTG 3’ |  |
|  | RV 5’ CGCATAACTCACAGGAACCAG 3’ |  |
| ***TLR9*** | FW 5’ CTATAACCGGAACTTCTGCCAG 3’ |  |
|  | RV 5’ CTGCTCTGTGTCAGGTGTG 3’ |  |
| ***TLR10*** | FW 5’ AGAGTTAAATGAAGAGTCTCGAGG 3’ |  |
|  | RV 5’ TGCCATCATAAAGGTTGTATCAATG 3’ |  |
| ***NLRP3*** | FW 5’ GATCTTCGCTGCGATCAACAG 3’ |  |
|  | RV 5’ CGTGCATTATCTGAACCCCAC 3’ |  |
| ***AIM2*** | FW 5’ TGGCAAAACGTCTTCAGGAGG 3’ |  |
|  | RV 5’ AGCTTGACTTAGTGGCTTTGG 3’ |  |
| ***DDX58(RIG1)*** | FW 5´ CTGGACCCTACCTACATCCTG 3’ |  |
|  | RV 5’ GGCATCCAAAAAGCCACGG 3’ |  |
| ***TMEM173 (STING)*** | FW 5´ CCAGAGCACACTCTCCGGTA 3’ |  |
|  | RV 5’ CGCATTTGGGAGGGAGTAGTA 3’ |  |
| ***MB21D1 (cGAS)*** | FW 5´ CACGAAGCCAAGACCTCCG 3’ |  |
|  | FW 5´ GTCGCACTTCAGTCTGAGCA 3’ |  |
| ***AGER*** | FW 5´ GTGTCCTTCCCAACGGCTC 3’ |  |
|  | FW 5´ ATTGCCTGGCACCGGAAAA 3’ |  |
| ***IFNL1F*** | FW 5´ AAC TGG GAA GGG CTG CCA CAT 3’ |  |
|  | FW 5´ GGA AGA CAG GAG AGC TGC AAC 3’ |  |
| ***IFNB1R*** | FW 5´ GCT TGG ATT CCT ACA AAG AAG 3’ |  |
|  | FW 5´ ATA GAT GGT CAA TGC GGC GTC 3’ |  |
| ***SREBF2*** | FW 5´ CCTGGGAGACATCGACGAGAT 3’ |  |
|  | FW 5´ TGAATGACCGTTGCACTGAAG 3’ |  |
| ***SQLE*** | FW 5´ GGCATTGCCACTTTCACCTAT 3’ |  |
|  | FW 5´ GGCCTGAGAGAATATCCGAGAAG 3’ |  |
| ***HMGCR*** | FW 5´ TGATTGACCTTTCCAGAGCAAG 3’ |  |
|  | FW 5´ CTAAAATTGCCATTCCACGAGC 3’ |  |
| ***ABCA1*** | FW 5´ ACCCACCCTATGAACAACATGA 3’ |  |
|  | FW 5´ GAGTCGGGTAACGGAAACAGG 3’ |  |
| ***MVD*** | FW 5´ CTCCCTGAGCGTCACTCTG 3’ |  |
|  | FW 5´ GGTCCTCGGTGAAGTCCTTG 3’ |  |
| ***ABCG1*** | FW 5´ ATTCAGGGACCTTTCCTATTCGG 3’ |  |
|  | FW 5´ CTCACCACTATTGAACTTCCCG 3’ |  |
| ***APOA1*** | FW 5´ CCCTGGGATCGAGTGAAGGA 3’ |  |
|  | FW 5´ CTGGGACACATAGTCTCTGCC 3’ |  |
| ***IDI1*** | FW 5´ AACACTAACCACCTCGACAAGC 3’ |  |
|  | FW 5´ AGACACTAAAAGCTCGATGCAA 3’ |  |
| ***APOL3*** | FW 5´ GGGACGAGTCTGGCCCTTA 3’ |  |
|  | FW 5´ TCAATCGGTCAATGCTGGTTG 3’ |  |
| ***APOL1*** | FW 5´ TGGACTACGGAAAGAAGTGGT 3’ |  |
|  | FW 5´ CCTCCTTCAATTTGTCAAGGCTT 3’ |  |
| ***LSS*** | FW 5´ GCACTGGACGGGTGATTATGG 3’ |  |
|  | FW 5´ TCTCTTCTCTGTATCCGGCTG 3’ |  |
| ***MVD*** | FW 5´ CTCCCTGAGCGTCACTCTG 3’  FW 5´ GGTCCTCGGTGAAGTCCTTG 3’ |  |
